# The social burden of antimicrobial resistance: what is it, how can we measure it, and why does it matter?

**DOI:** 10.1093/jacamr/dlae208

**Published:** 2025-03-10

**Authors:** Katherine Keenan, Juliana Silva Corrêa, Luechai Sringernyuang, Susan Nayiga, Clare I R Chandler

**Affiliations:** School of Geography and Sustainable Development, University of St Andrews, St Andrews, UK; Getulio Vargas Foundation, São Paulo School of Business Administration (EAESP FGV), São Paulo, Brazil; Getulio Vargas Foundation, São Paulo School of Business Administration (EAESP FGV), São Paulo, Brazil; Contemplative Education Center, Mahidol University, Nakhon Pathom, Thailand; Infectious Disease Research Collaboration, Kampala, Uganda; Department of Global Health and Development, London School of Hygiene and Tropical Medicine, London, UK; Department of Global Health and Development, London School of Hygiene and Tropical Medicine, London, UK

## Abstract

Antimicrobial resistance (AMR) is a growing global health threat, which is increasingly quantified in terms of its human health and economic burden. In this article, we highlight that for policy and planning purposes the social burden of AMR is as important to attend to as health and economic burdens, requiring systematic consideration and measurement of multiple dimensions. We provide a conceptual and empirical overview of four dimensions of the social burden of AMR: the distribution of AMR among and between populations; the lived experiences of AMR by patients and carers; how and by whom AMR interventions are shouldered; and how AMR can change society. We illustrate these dimensions through five case studies drawn from research projects in the UK, East Africa, Thailand and Brazil. Drawing on these insights, we discuss challenges and opportunities for documentation and measurement of AMR’s social burden going forward. Taking this seriously aligns with the consensus observation that to address AMR requires moving away from pathogen-based and siloed disciplinary perspectives and means embracing different forms of data and evidence from around the world. We propose an interdisciplinary engagement across researchers, policy makers and community stakeholders to arrive at agreed principles and metrics for future monitoring of the social burden. We need to tackle invisibility through lack of data by considering the social burden in design of AMR surveillance and research, includes mainstreaming social science data, and incorporating arts-based approaches to understanding AMR. Recognition, documentation and measurement of the social burdens of AMR will advance AMR approaches and help develop equitable solutions.

## Introduction

Antimicrobial resistance (AMR) describes a process whereby viruses, fungi, parasites and bacteria become resistant to the medicines designed to suppress them. The number of pathogens that have become resistant to antimicrobials appears to be rising globally, with a profound impact on human, animal and environmental health. For example, antibacterial resistant infections are estimated to have caused 1.1 million human deaths in 2021,^1^ and are forecasted to increase to 1.9 million by 2050.

Such quantitative estimates of the burden of AMR have become powerful motivators for research and policy to tackle the issue. Over the past 20 years, measurement has moved from a focus on bugs, to attributable deaths, to encompass economic costs. The World Health Organization’s 2001 strategy^2^ on AMR arose from the 1998 World Health Assembly declaration.^3^ The importance of clinical and economic burdens was highlighted across these calls to action, but the recommended measurement was of pathogens, through laboratory surveillance and reporting. At this time, a development frame^4^ meant that the consequences of AMR for the poorest and most vulnerable were recognized and the prevention of infection for these groups was highlighted as a priority. However, the launch coincided with a year of geopolitical change, including the 9/11 terrorist attack in New York, and the strategy failed to gain traction.^5^ Meanwhile, regional surveillance systems like the European EARS-Net, set up in 1997, began to enable governments to estimate how many deaths are attributable to antibacterial resistant infections in Europe^6^ and the USA.^7^ Having seen limited progress globally in the decade subsequent to the 2001 strategy launch, other ways to capture the significance of the AMR problem were put forward. The UK Government commissioned a report that estimated >700 000 annual deaths globally attributable to AMR^8^—including TB and malaria—and, importantly, expanded this analysis to economic estimates and future projections: by 2050 a potential cumulative cost to global economic output of 100 trillion USD and 10 million lives lost annually.^9^ The World Bank followed with a similar cost projection based also on macro-economic methodology.^10^ The 2019 and 2021 mortality estimates, also funded by the UK Government through the GRAM consortium, explored age and regional disparities, while highlighting the limited data for disaggregated analyses.^1,11^ Calculations of the economic burden of AMR, including the cost of treating resistant infections, combined with economic productivity losses are alarmingly high.^12,13^ There has been growing concern over the potential inequalities—albeit not well documented—in the AMR burden. The declaration agreed at the 2024 UN General Assembly level meeting notes the ‘profound socioeconomic challenges and financial hardships faced by people affected by AMR… and therefore affirm that all these people require integrated, people-centred prevention, diagnosis, treatment, management of side effects, and care…’ (paragraph 15^14^), and commits to reduce antibacterial resistance (ABR)-associated deaths by 10% by 2050. Achieving this will require addressing—and making tangible and legible—the social burden of drug resistance.

**Figure 1.**
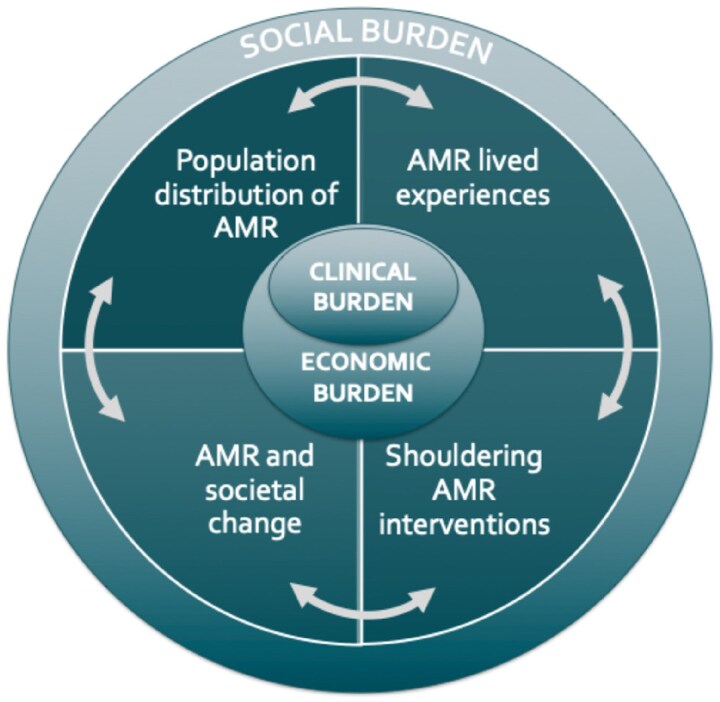
Four inter-related, multi-scalar dimensions of the social burden of AMR.

While efforts to measure and articulate the health and economic burdens of AMR have gained traction in quantitative form, the wider social impacts of AMR have been evoked more often in narrative form framed as, for example ‘the end of modern medicine’ or ‘antibiotic apocalypse’^15^ rather than being systematically studied and used to inform decisions. The growing awareness of the importance of social science insights for AMR is notable,^16^ and many organizations including the EU, the WHO and various National Action Plans call for greater attention to social impacts.^17^ However there is a need to unpack what ‘social’ means in this context: it is often envisioned as a barrier or facilitator to AMR interventions rather than a field that is being actively shaped by AMR. We highlight that the ‘social burdens’ of AMR should be attended to as closely as the AMR clinical and economic burdens, but that this attention may require different forms to render the burden legible and trackable. In economic terms, some of the aspects of social burden of AMR overlap with the notion of ‘social costs’ including costs to individuals and externalities. While these could be quantified and modelled, we argue for the need to make explicit the social burden in everyday terms. Any description of AMR burden is complicated by the fact that AMR is not a single condition, but a process that occurs across different microbial species, with different pathogenicity in different situations and with different transmission routes and differing adverse consequences of resistance. Nonetheless, we concur with those creating global unified estimates of clinical and economic burdens that there can be value in articulating an heuristic for focusing attention for political and programmatic action. In this article, we draw on prior research to propose four key inter-related dimensions of social burden of AMR (Figure 1), and suggest how they could be conceptualized and measured across multiple ecosystem domains.

## The population distribution of AMR

AMR microbes develop and spread among environment–animal–human interfaces in uneven ways as a result of the folding together of historical, ecological and biological processes.^18,19^ Human and animal surveillance data show geographic inequality in resistance and AMR mortality burden at both regional and national scales,^1,11,20,21^ and this is typically higher in resource-poor and more socioeconomically unequal societies^21^ and in the global South.^11,21^ At sub-national levels, there is sparse reliable epidemiological evidence in many countries. Higher AMR environmental loads have been found in urban areas with higher population density,^22,23^ where water sources or environments may be contaminated,^24^ including by wet season flooding and animal contact.^23,25^ At both area- and individual-levels, lower socioeconomic status and poverty including overcrowding, homelessness, lower income and lower education^25–27^ are also associated with higher AMR rates. Ecological studies find that differences in antibiotic usage do not fully account for national inequalities, and point to certain forms of political, economic and governance systems as drivers of higher AMR.^21,28^

The burden of AMR infections in humans is also differentially distributed according to key societal stratifiers, such as age, gender, race and socioeconomic status.^29–31^ For example, AMR-related mortality peaks in the neonatal period and in the very old,^1,32^ and advanced age is a common risk factor, intersecting with gender in complex ways.^23,30^ Global population ageing and the projected expansion of (multi) morbidity presents additional risks through clinical vulnerability and increased contact with healthcare settings^33^ (see Case study 1). These observed inequalities are the result of layered vulnerabilities, exposures, treatments and opportunities for care, a complex process which requires careful research incorporating a range of different types of data. There is also a need to attend to the production of intersectional inequalities in antibiotic use and AMR risk which operate through combinations of social disadvantage^34^ and result from wider historical and social processes.^18^ Gender is one such intersectional lens that has received most recent attention. Men and women are probably differently exposed to infection, drug resistance, antibiotics and also have differential access to care. For example, women and girls in low resource settings may be at increased risk of (drug-resistant) infection due to their menstrual hygiene needs yet have poorer access to sanitation, and have more limited decision-making power to seek care.^35^

The lack of linked data on AMR cases including demographic and social dimensions is a clear hinderance to research on this topic.^29,30^ Another major challenge to understanding the nature of AMR inequalities is that most AMR data is itself subject to various forms of selection bias making it difficult to distinguish real social inequalities from data artefacts. Most human AMR studies are subject to ‘isolate bias’, being based on non-randomly sampled bacterial isolates from hospital settings. The social determinants of health and health care access play into this process of selection bias. Individual and community vulnerability to develop a serious (resistant) infection, the propensity for that person to seek help in a hospital setting where diagnostic cultures are performed and shared for research and the ability to pay for such services illustrate profound limitations of existing data to represent social stratification. Joined up efforts are required to establish core data assemblages including demographics and socioeconomic factors, recognize bias transparently and include additional sources of (qualitative) data to enable a nuanced interpretation that takes account of pathways of vulnerability, exposure and treatment.

**Case study 1: Unequal population distribution of AMR: co-occurrence of cancer and AMR infections**

AMR infections intersect with age-related diseases such as cancer^36,37^ in complex, synergistic ways. Infection is a major cause of death and complications in cancer patients,^38,39^ associated with ∼60% of cancer deaths.^39^ Resistant infections such as ESBL-producing bacteraemia and VRE are an increasing problem, and are more prevalent among cancer patients in intensive care and those receiving antibiotics.^40^ On larger scales, this threatens to reverse cancer survival gains made in the 20th century.

Cancer and AMR infections co-occur in a syndemic fashion^41^ combining to exacerbate the risks and consequences of each other. Cancer survival is dependent on surgical and chemotherapeutic interventions which increase susceptibility to infection, while simultaneously increasing exposure to healthcare settings where resistant bacterial and fungal infections thrive.^42,43^ Antibiotics act as a scaffold for cancer care and are used prophylactically to reduce the risks of infection. This higher antibiotic use to treat infections and to prophylactically manage the risks of interventions fuels further drug resistance.

The age and socioeconomic gradient of cancer risk and survival^44^ means that the cancer–AMR syndemic will create a disproportionate mortality burden for older, socioeconomically deprived groups. However early onset cancer is becoming more common,^45^ a manifestation of risk factors such as obesity and environmental pollution in younger generations. Therefore, in the future, AMR infections could have even greater impact on cancer patients in mid-life. The social burden of AMR for mid-life cancer patients looks different because they are typically in more active caring and labour force roles compared with older people. Quantifiable economic and social burdens include higher costs of treating both cancer and resistant infections, longer hospital stays, caring burden, loss of productivity and quality of life. In countries with fewer resources these burdens are more often borne by individuals, households and communities. Thus, the cancer–AMR infection syndemic may create double or triple burdens for affected individuals and their social networks.

## AMR lived experiences

Beyond high-level statistics, the lived experiences of those dealing with AMR tell of the (often hidden) social burden, which extends through socio-material networks. The narratives of patients struggling to find care for chronic drug-resistant infections show adverse effects on many life domains including mental health, work, family and social lives.^46^ Accounts of pathways to care for those with AMR infections highlight how social disadvantage makes accessing appropriate care more complex and time consuming, especially within fragmented and under-resourced health care systems in the global South.^47^ These issues are under-documented, and the ‘collateral damage’ of AMR infections on carers, and how that falls unequally, has rarely been explored (see Case study 2). The intersectional gender and socioeconomic disparities in informal care-giving^48,49^ suggest that lived experiences of AMR are unequal.

Various organizations including the WHO have recently initiated campaigns to make visible the accounts of ‘AMR survivors’ (https://www.who.int/groups/task-force-of-amr-survivors, https://amrnarrative.org/) and use this as tool for political engagement and activism. These so far have taken a patient-centric perspective, which could be extended to better understand the experiences of those caring for the sick, whether informally or in formal care systems, for example when trying to treat AMR infections and manage the risks of medical interventions with a dwindling stock of effective antimicrobials. Beyond human health, the experiences of farmers facing the consequences of AMR for their animals and livelihoods have begun to be documented^50^ but deserve more attention, going further than surveys of knowledge and attitudes.^51^

Understanding what it takes to manage the burden of AMR—to care, survive and thrive when antimicrobials are not working—can usefully be inferred from other infection scenarios for which there is no quick-fix management. Thus, the initial stages of COVID, HIV and Ebola provide a window into the webs of care and control that characterized these infections in different ways. For many who live with drug-resistant TB, this is already a reality, as well as for cystic fibrosis patients for whom catching infections can spell very serious consequences.^52^ Bedridden patients and their carers form one group who are now living with the effects of AMR, differentiated by circumstance (Case study 2). To capture and communicate this burden is to require documentation or measurement that can incorporate the wider AMR assemblage, considering the multitude of actors and structures and how they are inter-related. This moves beyond biomedical accounts of AMR, which foreground counting of pathogens and drugs, by considering people and environments as co-constituents in AMR processes and burdens.

**Case study 2: AMR lived experiences: bedridden life and care in peri-urban Thailand**

Aunt C welcomes us into her sister-in-law’s home—she has been waiting for us. Amidst boxes of dialysis solution and the vestiges of what must once have been a beauty salon, we find Uncle T lying on his side with his back to the door. A big man in his 60s, not long ago able to drive a small truck for his living in this peri-urban area near Bangkok, Thailand, he now closes his eyes as if wanting to cut himself off from the world. The pair—now staying with Uncle T’s younger sister for support—are exhausted from the ordeal of the past couple of months. First hospitalized for a month at the provincial hospital after a herniated disc caused Uncle T to lose feeling in his lower body, he was then referred to the regional hospital where he remained bedridden for another month. Aunt C has put her own work on hold to look after Uncle T in a health system that, while attempting to provide Universal Health Coverage, also relies on family to deliver care.^53^ In his time at the regional hospital, Uncle T developed painful bedsores. By the time he arrived home, his sister was so concerned with the size of these sores that she called the nurses at the local health centre for help. But the size—bigger than two palms of the hand—was too big for even them to manage and they asked for him to be seen back at the hospital. However, the hospital were unable to see him until his return appointment date, leaving his wife and sister in a conundrum, having been told to ‘help him themselves’. Aunt C explained to the nurse, ‘I was thinking for a long time whether I should call you or not… but I could not do anything else… so then I called.’ The nurse cleaned the sore as best she could and left behind some cleaning kits. But Uncle T’s worsening condition led to hospital readmission after 2 days, his wife following him to provide care at the hospital. In the course of this stay, Uncle T developed an infection that turned out to be drug resistant. Visiting him on the ward, in the zone allocated for AMR cases, we found him looking thin and with a frowning expression. His wife explained the events leading to his infection, which appeared to have been introduced with a Foley catheter. Now, she was responsible not only for caring for him on the ward but caring for the drug-resistant microbes that could be spread to other patients, ‘The doctor said to me that Uncle is infected with a very lethal and dangerous germ. That I needed to use all the things (signalling to a box with a gown, gloves, bottle of alcohol gel, that sat next to the red label “AMR” at the foot of Uncle T’s bed) provided here before and after touching my husband.’ Aunt C sighed. It was hard to follow this advice: caring for her husband did not align with the wearing of gowns and gloves. After a few more days, Uncle T was not able to survive the infection. Three stays of a month each in hospital had drained him and his closest family. Caring for drug resistance is a burden shouldered not only by the health system but the family, an often invisible burden in a setting where despite efforts of Universal Health Coverage, many still fall through the cracks.^53,54^

## Shouldering AMR interventions

Typically, AMR interventions focus either on reducing and targeting antimicrobial use to prevent or slow the rate at which pathogens develop resistance, or on reducing the transmission of pathogens between humans or between animals and humans. Antimicrobial use interventions—currently under the umbrella of ‘stewardship’—often rely on a three-pronged approach of surveillance, restriction and correction,^55^ implemented through measurement, regulation and educational campaigns.^56^ Such campaigns intend to change antimicrobial use behaviour by creating subjects who will choose to use these medicines with a particular form of rationality.^57^ Likewise, interventions to reduce transmission—whether under the umbrella of infection control or biosecurity—also rely on surveillance but coupled with separation and containment activities that are devolved to the responsibility of individual caregivers or farmers.^58^ The logics of such interventions also take on social forms as moral imperatives that intersect with social categories of cleanliness and who cleans. In both cases—making antimicrobial use rational and making microbes containable—the potential for interventions to be unevenly shouldered by those requested to implement must be considered (see Case study 3).

AMR interventions are also often expected to be taken on by particular groups: prescribers, drug sellers, farmers and sick patients; who struggle to implement recommendations and navigate commercial, ethical and real-world pressures.^59–61^ Moreover, in promoting optimal antibiotic use, they often seek to correct perceived irrational behaviours, including ‘informal care’ and use of drug sellers. Such providers operate disproportionately in low-resource settings in Asia and Sub-Saharan Africa to plug gaps in public healthcare provision and increase access to medicines for otherwise poorly serviced populations,^62^ which raises issues of equity. More recently there has been renewed attention to upstream drivers of infection and transmission such as vaccination^63^ and water and sanitation,^58^ which can locate responsibility at supra-individual levels.

Treatment for AMR infections is already socially stratified and could become more so. Access to diagnostics to target antibiotic therapies shows huge global disparities.^64^ As first-line treatments for many common infections such as urinary tract infection and syphilis become ineffective, the accessibility of more expensive second- and third-line treatments is jointly determined by market forces, health infrastructures and wealth.^65^ The high development costs of new antibiotics^66^ makes prioritizing equitable access to new therapies a critical policy challenge.

**Case study 3: Shouldering AMR interventions: lives and livelihoods in rural Uganda**

In our research on medicines and health in rural Eastern Uganda over the last 15 years, we have observed the effects of efforts to restrict access to antibiotics without a prescription as well as of efforts to restrict livestock–human contact in domestic spaces. In particular, we have observed the significant burden of these restrictions for residents whose resources are minimal, in an area with chronically inadequate infrastructure and multiple dimensions of insecurity including personal, economic and climate. Grace, a 40-year-old mother of four, seems the epitome of the entrepreneurial spirit cultivated in Ugandan citizens. She spends her time taking care of her extended family, piecing together financial opportunities and finding ways to manage their various ailments, injuries and more serious health concerns.^67^ Financially, Grace rarely breaks even, having to take out loans to pay start-up costs for small-scale initiatives and to pay for school costs. Health issues place an additional cost burden, especially as the free care provided at the local health centre is often unavailable, either because of health worker absence or a lack of medicines.^68^ Unlike families with a more secure income, who might seek care from private clinics where they are first examined by trained professionals (usually clinical officers or enrolled nurses) and then prescribed medicine, Grace aligns with most residents in her area, for whom drug shops are the core source of care.^69^ From these vendors (often nursing assistants), she can purchase smaller doses of medicines—even one antibiotic capsule at a time—in line with the funds she can gather. She, and the drug sellers, are aware that this is deemed inappropriate. Indeed, one of the many roles Grace plays is as a volunteer in the Village Health Team. However, along with many others resorting to purchasing antimicrobials over the counter (OTC), she is unable to access further information about appropriate medicine use. This is in part because drug shops and their vendors—as well as community health workers—are deemed outside of the legitimate sphere for antimicrobial provision as Class C drugs (Class C drugs in Uganda are those that are recommended for sale OTC, and without prescription, in so-called ‘Class C drug shops'; these include moderate painkillers but exclude most forms of antibiotics, https://faolex.fao.org/docs/pdf/uga104984.pdf). Professional authorities are concerned that information on how to use these medicines could imply endorsement of their use OTC, and thus could fuel inappropriate use.^70^ Thus, efforts to contain antibiotic use to prescribed contexts, coupled with inadequate services at government health facilities, find Grace and many others unable to access antimicrobials as recommended in clinical guidelines. These realities confront implicit intervention narratives of the public’s ignorance and irrationality.^57^ Grace also shows us the challenges of implementing biosecurity guidelines as imagined in One Health policies. As one way to generate income, Grace agreed to look after five goats for a relative in the city. She was anxious about leaving the homestead for fear of theft of these animals, and indeed she returned one day to find the small store where she kept them empty. The consequences were severe: the goats’ owner reported the loss to the police and Grace was arrested and jailed until funds could be found to pay for bail. After this, despite being familiar with the ongoing guidance to avoid cross-species disease transmission by keeping livestock separate from the domestic sphere, Grace decided to keep subsequent livestock in her house overnight, where the family also sleeps, to ensure the safety of the animals as well as herself. However, she is aware this decision comes with risks, given the exemplary role she is expected to play as part of the Village Health Team. Despite working hard to be the kind of citizen the state defines as successful, Grace finds herself at risk from multiple dimensions as she struggles to keep her family’s finances and health afloat. Her situation reminds us of the uneven way in which interventions that intend to reduce the drivers of AMR can have an impact, particularly for those with the most limited means.

## AMR and societal change

To explore the changes AMR can make to society we must first appreciate how the mass introduction of antimicrobials dramatically altered 20th century ecosystems to the extent that these substances became taken for granted as part of our sociotechnical infrastructure.^71^ Antibiotics revolutionized infectious disease treatment, contributing to steep mortality declines, particularly for infants.^72^ This catalysed the demographic transition, stimulating life expectancy rise, fertility decline and global population ageing. Antibiotic treatments promoted epidemiological and health transitions, i.e. a shift in the causes of death from infectious to non-communicable conditions and longer survival with chronic conditions.^73^ Under current forecasts, AMR will further drive demographic and health transformations, and these could reshape disease paradigms, prevention efforts and future assessment of risk. The re-emergence of difficult-to-treat infections threatens life expectancy gains, at the same time as the world’s population ages, and there is evidence that life course population health in high income settings is declining.^74^ Further, the interaction of non-communicable diseases with complex infection (see Case study 1) and long-term chronic living of AMR patients challenges the notion of communicable infections as short term, and non-communicable disease as chronic. The rise of complex chronic infectious multimorbidities demands more flexible health systems that can move beyond the outdated single disease paradigm.

Over the 20th century, food and farming systems have also become reliant on antimicrobials to control infection and promote production.^75^ Livestock production accounts for more than three-quarters of global antimicrobials sales, which has fuelled rises in resistance in animals and the environment.^76^ Although there is still uncertainty about the relative impact of agricultural antibiotic use on human health compared with other drivers of the clinical burden of resistance,^77^ concern about ecosystem spillovers have led to environmental antibiotic stewardship policies, notably in richer nations. But the commercial determinants of health, consumer demand and food security concerns clash with agricultural governance and infrastructural change. Some suggest that ecological consequences of intensive farming (including climate change and increased AMR) could necessitate a large-scale dietary transition to reduced protein consumption,^78^ with consequences for population health and livelihoods. Our AMR response therefore brings up ethical tensions between the needs of countries and populations, some of whom are more or less dependent on intensive farming and antimicrobials for current and future generations.^79^ These political economy tensions can be illustrated with a case study of the Brazilian agricultural sector (Case study 4).

**Figure 2.**
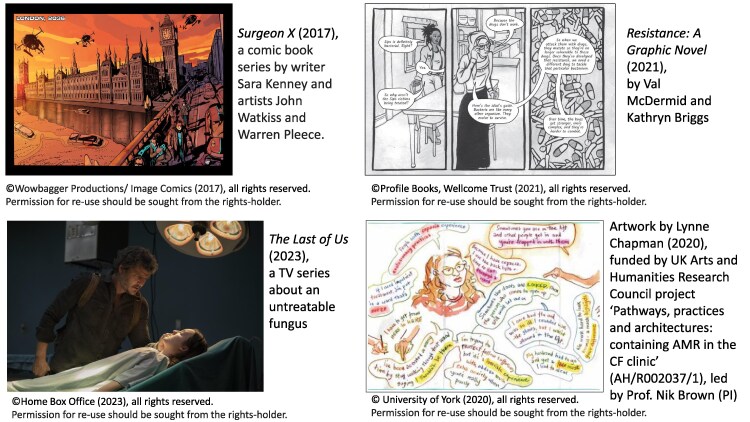
Representations of AMR in art, literature and media.

The potentially profound changes that AMR could lead to in societies has been presented evocatively through the arts and fiction, in particular, with the rise of science fiction and pandemic anxieties.^80^ Figure 2 shows some recent examples from science fiction literature and the media which all build on the understanding that antimicrobials are an intrinsic part of our societal infrastructure, bringing order and controlling risk in our mastery of nature. With their diminishing power, post-antibiotic worlds deploy narratives of apocalypse, catastrophe,^81^ evoke regression to dark, chaotic histories (‘return to the dark ages’, ‘coming plague’) and use military metaphors for battles against outbreaks of untreatable ‘superbugs’.^82^ However, recently there has been a turn towards imagining microbial co-existence.^15,83^ This optimistic space allows us to conceive our future differently and analysing the changes that AMR promotes in society. Just as antimicrobials have come to be seen as a ‘quick fix’ for care, productivity, hygiene and inequality,^84^ we have the opportunity to discuss how to go from quick fix to long-term sustainable design interventions that can positively mitigate the social burden. Intersections with climate change, and the One-Health paradigm demands greater policy attention and understanding of connections between human, animal and environmental domains, promoting a shift from anthrocentrism to ecosystem-based approaches.

**Case study 4: AMR and societal change: perspectives from the agricultural sector in Brazil**

In Brazil, the agricultural sector constitutes 24% of the nation’s Gross Domestic Product, making the country a global leader in the export of beef, pork and poultry.^85^ The expansion of intensive animal production has been accompanied by increased use of antibiotics for illness treatment and prevention in confined animals, and growth promotion. The escalation of resistance rates has led to gradual reforms in the use of antibiotics and other performance-enhancing additives.^85^

A qualitative study conducted by J.C.^86^ with stakeholders in agricultural policy offers insights into the societal impacts of AMR, particularly through the pressures exerted on the agricultural production process and its governance. Interviewees underscored the imperative for Brazil to demonstrate adherence to the global AMR agenda to preserve its standing in the international food export market. However, beyond formal commitments, the narratives revealed the practical challenges of implementing actual changes amidst conflicting pressures from industry, multilateral organizations and governments. While stakeholders did acknowledge the AMR problem, discussions regarding whether, when and how to restrict the use of AB as growth promoters highlighted significant tensions between Brazilian agricultural stakeholders and international AMR policy reformers. In their horizon of expectations, more structural changes in intensive farming are still far from happening. The implementation costs of interventions and the potential increase in food prices for consumers could prevent people from accessing animal protein, which was perceived to be more important at this moment for Brazil:

‘We always have to remember that we live in a country where access to food is different from a reality like Denmark, for example. (…) They (Denmark) can afford to buy chicken that uses antibiotics only responsibly and that has a certification for this, but this all has costs to implement it.’ (Ministry of Agriculture)

Stakeholders perceived the influence of top-down national governance as limited, suggesting that more substantial long-term changes could emerge from the ground up, with consumer choices exerting market pressures that compel industries to adopt more responsible production practices. As one stakeholder noted, ‘This is the work of society as a whole to realize the importance of antibiotics, which is a tool that we depend on. Sometimes it matters more than the work of the Ministry of Agriculture, the Ministry of Health, because our strength is great, but is limited.’

This example highlights some potential ways AMR will force societies to change the way they produce, regulate and consume in a context economically and nutritionally dependent on meat. How do global governance mechanisms account for the local challenges faced in implementing effective AB stewardship considering the economic, social and political factors? Will solutions be driven by statutory reforms or led by consumer pressures? Are there going to be structural reforms or incremental changes in the way production is developed,^75^ and how will these changes be shouldered by different communities and places?

## Interconnected social burdens

As Figure 1 shows, the four dimensions of social burden discussed above interconnect, helping to drive and exacerbate one other. Drawing on mixed-methods evidence assembled from East Africa, Case study 5 illustrates reciprocal relations between multidimensional poverty and antibacterial resistance.

**Case study 5: Interconnected social burdens: poverty and AMR in East Africa**

Recent One-Health studies in East Africa have illuminated systems of AMR risk and household poverty in contexts of extremely high levels of drug resistance.^23,47,87^ Aspects of poverty such as contaminated water and poor sanitation, chronic illness and lower education interact with environmental exposures such as contact with animals, urban environments and seasonal flooding to create high-risk AMR contexts.^23,25^ This probably happens through multiple cyclic mechanisms: higher transmission of infection that drives demand for antibiotics, in turn fuelling further driving resistance. Regardless of unequal risks of AMR infection, everyone faces immense structural challenges in accessing appropriate diagnostics, care and medicines.^59,87^ Care journeys are complex and expensive amid an often-confusing landscape of different providers.^47^ This drains time and material resources from already ill patients. One patient describes their fifth attempt to get care with frustration and helplessness:

‘When the sickness continued, I [visited another] doctor … He tested me, gave me medicine, and injected me six times … Then he took my urine samples … He told me that the medicine he was injecting couldn’t cure it. He then brought another type of medicine … [but] when the dose was over … I started feeling like the disease had come back. Then I wondered how I was, if I had gone everywhere and the disease was failing, now where was I to go!’ (Male patient age 28, Mbarara).^47^

In turn, AMR infections materially affect livelihoods through various mechanisms: for example, time seeking care is time off work, the costs of repeat treatments and inpatient care for those suffering.

### Conclusion: making the social burden of AMR count

How can the social burdens of AMR be made legible alongside the clinical and economic burdens that have been made to count through their more quantifiable nature?^88^ There are numerous challenges to overcome, which we propose are tackled using an equitable interdisciplinary approach, which does not privilege one type of data or knowledge. The first challenge is invisibility through lack of data, caused by little consideration of social burdens in the design of AMR surveillance and research. On the data front, a good first step would be to measure characteristics of units other than pathogens: people, animals and ecosystems. Current AMR surveillance efforts rarely include detail about the people or the contexts the biological samples are derived from, so occlude the social burden by design. We agree with calls for people-focused approaches^17^ that push for AMR data standards that include demographics^30^ and other social data. We should also design future AMR studies to take account of intersectional complexity in historical context, and how multiple social factors interact to shape AMR outcomes. The second challenge is the well-established issue of hierarchies of knowledge, which have long stood in the way of developing methods to display insights from qualitative research such that they are taken with equal seriousness as quantitative data. Mainstreaming social science and data science approaches (e.g. data linkage, qualitative and participatory techniques) would illuminate contextual complexities of social burden. It would also drive a better understanding of systems, upstream drivers of burden and how to intervene appropriately.^89^ Having an empirical view on the issue would bring more nuanced attention to scale, perspective, inequality and justice within AMR.^78^ But quantification and qualification using traditional written forms of social scientific data might not gain sufficient attention. We propose there is scope to incorporate with serious intent the impactful artistic and journalistic approaches to communicate about AMR (e.g. Figure 2) to diverse audiences. This raises a third challenge, to work together across disciplines to embrace skillsets not just as translational but as foundational to our understanding—not just communication—of the AMR problem. Such approaches have the potential to bring much needed creativity to help imagine solutions. Furthermore, these forms should and could be included in monitoring to broaden evidence of burden beyond the usual metrics.

In this paper, we have shone a light on multiple dimensions of AMR social burdens, but these are not exhaustive. Our heuristic should be expanded upon by those with different experiences and perspectives as we move forward with the ambitious global plans for interconnected action on AMR, supporting further development of a research agenda and evidence base. Recognition, documentation and measurement of the social burdens of AMR will advance AMR approaches and help develop equitable solutions.

## References

[1] GBD 2021 Antimicrobial Resistance Collaborators . Global burden of bacterial antimicrobial resistance 1990–2021: a systematic analysis with forecasts to 2050. Lancet 2024; 404: 1199–226. 10.1016/S0140-6736(24)01867-1 39299261 PMC11718157

[2] World Health Organisation . 2001. WHO Global Strategy for Containment of Antimicrobial Resistance. https://www.who.int/publications-detail-redirect/who-global-strategy-for-containment-of-antimicrobial-resistance

[3] World Health Organisation . 1998. Fifty-First World Health Assembly. Geneva 11-16 May 1998. Resolutions and Decisions Annexes. https://iris.who.int/bitstream/handle/10665/258896/WHA51-1998-REC-1-eng.pdf?sequence=1

[4] Wernli D, Jørgensen PS, Morel CM et al Mapping global policy discourse on antimicrobial resistance. BMJ Glob Health 2017; 2: e000378. 10.1136/bmjgh-2017-000378 PMC5717922 29225939

[5] Overton K, Fortané N, Broom A et al Waves of attention: patterns and themes of international antimicrobial resistance reports, 1945–2020. BMJ Glob Health 2021; 6: e006909. 10.1136/bmjgh-2021-006909 PMC8573652 34740914

[6] Cassini A, Högberg LD, Plachouras D et al Attributable deaths and disability-adjusted life-years caused by infections with antibiotic-resistant bacteria in the EU and the European economic area in 2015: a population-level modelling analysis. Lancet Infect Dis 2019; 19: 56–66. 10.1016/S1473-3099(18)30605-4 30409683 PMC6300481

[7] Centers for Disease Control and Prevention . 2013. Antibiotic Resistance Threats in the United States, 2013. https://www.cdc.gov/antimicrobial-resistance/media/pdfs/ar-threats-2013-508.pdf

[8] O’Neill J . 2014. Antimicrobial Resistance: Tackling a Crisis for the Health and Wealth of Nations. https://amr-review.org/sites/default/files/AMR%20Review%20Paper%20-%20Tackling%20a%20crisis%20for%20the%20health%20and%20wealth%20of%20nations_1.pdf

[9] O’Neill J . Tackling Drug-Resistant Infections Globally: Final Report and Recommendations. Government of the United Kingdom; 2016. https://apo.org.au/node/63983

[10] Jonas OB, Irwin A, Berthe FCJ et al Drug-Resistant Infections: A Threat to Our Economic Future (Vol. 2). Final Report (English). World Bank Group, 2017. http://documents.worldbank.org/curated/en/323311493396993758/final-report

[11] Antimicrobial Resistance Collaborators . Global burden of bacterial antimicrobial resistance in 2019: a systematic analysis. Lancet 2022; 399: 629–55. 10.1016/S0140-6736(21)02724-0 35065702 PMC8841637

[12] Ahmad M, Khan AU. Global economic impact of antibiotic resistance: a review. J Glob Antimicrob Resist 2019; 19: 313–6. 10.1016/j.jgar.2019.05.024 31176071

[13] Morel CM, Alm RA, Årdal C et al A one health framework to estimate the cost of antimicrobial resistance. Antimicrob Resist Infect Control 2020; 9: 187. 10.1186/s13756-020-00822-6 33243302 PMC7689633

[14] United Nations General Assembly . 2024. Political Declaration of the High-Level Meeting on Antimicrobial Resistance. www.un.org/pga/wp-content/uploads/sites/108/2024/09/FINAL-Text-AMR-to-PGA.pdf

[15] Servitje L . Gaming the apocalypse in the time of antibiotic resistance. Osiris 2019; 34: 316–37. 10.1086/704048

[16] Tompson AC, Chandler CIR. Addressing antibiotic use: insights from social science around the world. A report collated with social scientists of the Antimicrobials in Society Hub. London School of Hygiene and Tropical Medicine, 2021. 10.17037/PUBS.04659562

[17] World Health Organization . 2024. People-Centred Approach to Tackling Antimicrobial Resistance: Key Principle of the Roadmap on Antimicrobial Resistance for the WHO European Region 2023–2030. WHO Regional Office for Europe. https://iris.who.int/handle/10665/376459

[18] Hinchliffe S . Postcolonial global health, post-colony microbes and antimicrobial resistance. Theory Cult Soc 2022; 39: 145–68. 10.1177/0263276420981606

[19] Landecker H . Life as aftermath: social theory for an age of anthropogenic biology. Sci Technol Hum Values 2024. 10.1177/01622439241233946

[20] World Health Organisation . 2022. Global Antimicrobial Resistance and Use Surveillance System (GLASS) Report: 2022. https://www.who.int/publications-detail-redirect/9789240062702

[21] Allel K, Day L, Hamilton A et al Global antimicrobial-resistance drivers: an ecological country-level study at the human–animal interface. Lancet Planet Health 2023; 7: e291–303. 10.1016/S2542-5196(23)00026-8 37019570

[22] Elder FCT, Proctor K, Barden R et al Spatiotemporal profiling of antibiotics and resistance genes in a river catchment: human population as the main driver of antibiotic and antibiotic resistance gene presence in the environment. Water Res 2021; 203: 117533. 10.1016/j.watres.2021.117533 34416649

[23] Cocker D, Chidziwisano K, Mphasa M et al Investigating One Health risks for human colonisation with extended spectrum β-lactamase-producing *Escherichia coli* and *Klebsiella pneumoniae* in Malawian households: a longitudinal cohort study. Lancet Microbe 2023; 4: e534–43. 10.1016/S2666-5247(23)00062-9 37207684 PMC10319635

[24] Nolan TM, Reynolds LJ, Sala-Comorera L et al Land use as a critical determinant of faecal and antimicrobial resistance gene pollution in riverine systems. Sci Total Environ 2023; 871: 162052. 10.1016/j.scitotenv.2023.162052 36758688

[25] Keenan K, Papathomas M, Mshana SE et al Evidencing the intersection of environmental, socioeconomic, behavioural and demographic drivers of antibacterial resistance in East Africa. 2024. 10.2139/ssrn.4724376.

[26] Alividza V, Mariano V, Ahmad R et al Investigating the impact of poverty on colonization and infection with drug-resistant organisms in humans: a systematic review. Infect Dis Poverty 2018; 7: 76. 10.1186/s40249-018-0459-7 30115132 PMC6097281

[27] McCowan C, Bakhshi A, McConnachie A et al *E. coli* bacteraemia and antimicrobial resistance following antimicrobial prescribing for urinary tract infection in the community. BMC Infect Dis 2022; 22: 805. 10.1186/s12879-022-07768-7 36307776 PMC9621144

[28] Collignon P, Beggs JJ, Walsh TR et al Anthropological and socioeconomic factors contributing to global antimicrobial resistance: a univariate and multivariable analysis. Lancet Planet Health 2018; 2: e398–405. 10.1016/S2542-5196(18)30186-4 30177008

[29] Blackmon S, Avendano E, Nirmala N et al Socioeconomic status and the risk for colonization or infection with priority bacterial pathogens: a global evidence map. *medRxiv* 24306293. 10.1101/2024.04.24.24306293. 24 April 2024 PMC12103885 39653050

[30] Waterlow NR, Cooper BS, Robotham JV et al Antimicrobial resistance prevalence in bloodstream infection in 29 European countries by age and sex: an observational study. PLoS Med 2024; 21: e1004301. 10.1371/journal.pmed.1004301 38484006 PMC10939247

[31] World Health Organization . 2018. Tackling Antimicrobial Resistance (AMR) Together: Working Paper 5.0: Enhancing the Focus on Gender and Equity. https://apps.who.int/iris/handle/10665/336977

[32] European Antimicrobial Resistance Collaborators . The burden of bacterial antimicrobial resistance in the WHO European region in 2019: a cross-country systematic analysis. Lancet Public Health 2022; 7: e897–913. 10.1016/S2468-2667(22)00225-0 36244350 PMC9630253

[33] Abebe F, Schneider M, Asrat B et al Multimorbidity of chronic non-communicable diseases in low- and middle-income countries: a scoping review. J Comorb 2020: 10. 10.1177/2235042X20961919. PMC7573723 33117722

[34] Gautron JMC, Tu Thanh G, Barasa V et al Using intersectionality to study gender and antimicrobial resistance in low- and middle-income countries. Health Policy Plan 2023; 38: 1017–32. 10.1093/heapol/czad054 37599460 PMC10566319

[35] World Health Organisation . 2024. Addressing Gender Inequalities in National Action Plans on Antimicrobial Resistance: Guidance to Complement the People-Centred Approach. https://iris.who.int/bitstream/handle/10665/378639/9789240097278-eng.pdf?sequence=1

[36] Clegg LX, Reichman ME, Miller BA et al Impact of socioeconomic status on cancer incidence and stage at diagnosis: selected findings from the surveillance, epidemiology, and end results: national longitudinal mortality study. Cancer Causes Control 2009; 20: 417–35. 10.1007/s10552-008-9256-0 19002764 PMC2711979

[37] Hastert TA, Beresford SA, Sheppard L et al Disparities in cancer incidence and mortality by area-level socioeconomic status: a multilevel analysis. J Epidemiol Community Health 2015; 69: 168–76. 10.1136/jech-2014-204417 25288143

[38] Elhadi M, Khaled A, Msherghi A. Infectious diseases as a cause of death among cancer patients: a trend analysis and population-based study of outcome in the United States based on the surveillance, epidemiology, and end results database. Infect Agent Cancer 2021; 16: 72. 10.1186/s13027-021-00413-z 34972537 PMC8719405

[39] Zembower TR . Epidemiology of infections in cancer patients. In: Stosor V, Zembower TR, eds. Infectious Complications in Cancer Patients. Cancer Treatment and Research: Springer International Publishing, 2014; 43–89. 10.1007/978-3-319-04220-6_2 PMC7120867 24706221

[40] Montassier E, Batard E, Gastinne T et al Recent changes in bacteremia in patients with cancer: a systematic review of epidemiology and antibiotic resistance. Eur J Clin Microbiol Infect Dis 2013; 32: 841–50. 10.1007/s10096-013-1819-7 23354675

[41] Singer M, Bulled N, Ostrach B et al Syndemics and the biosocial conception of health. Lancet 2017; 389: 941–50. 10.1016/S0140-6736(17)30003-X 28271845

[42] Teillant A, Gandra S, Barter D et al Potential burden of antibiotic resistance on surgery and cancer chemotherapy antibiotic prophylaxis in the USA: a literature review and modelling study. Lancet Infect Dis 2015; 15: 1429–37. 10.1016/S1473-3099(15)00270-4 26482597

[43] Perez F, Adachi J, Bonomo RA. Antibiotic-resistant Gram-negative bacterial infections in patients with cancer. Clin Infect Dis 2014; 59 Suppl 5: S335–9. 10.1093/cid/ciu612 25352627 PMC4303050

[44] Vaccarella S, Georges D, Bray F et al Socioeconomic inequalities in cancer mortality between and within countries in Europe: a population-based study. Lancet Reg Health Eur 2023; 25: 100551. 10.1016/j.lanepe.2022.100551 36818237 PMC9929598

[45] Ugai T, Sasamoto N, Lee HY et al Is early-onset cancer an emerging global epidemic? Current evidence and future implications. Nat Rev Clin Oncol 2022; 19: 656–73. 10.1038/s41571-022-00672-8 36068272 PMC9509459

[46] Whittaker A, Do TT, Davis MDM et al AMR survivors? Chronic living with antimicrobial resistant infections. Glob Public Health 2023; 18: 2217445. 10.1080/17441692.2023.2217445 37272390

[47] Keenan K, Fredricks KJ, Al Ahad MA et al Unravelling patient pathways in the context of antibacterial resistance in East Africa. BMC Infect Dis 2023; 23: 414. 10.1186/s12879-023-08392-9 37337134 PMC10278291

[48] Schatz E, Seeley J. Gender, ageing and carework in East and Southern Africa: a review. Glob Public Health 2015; 10: 1185–200. 10.1080/17441692.2015.1035664 25947225 PMC4888771

[49] Tokunaga M, Hashimoto H. The socioeconomic within-gender gap in informal caregiving among middle-aged women: evidence from a Japanese nationwide survey. Soc Sci Med 2017; 173: 48–53. 10.1016/j.socscimed.2016.11.037 27918891

[50] Kayendeke M, Denyer-Willis L, Nayiga S et al Pharmaceuticalised livelihoods: antibiotics and the rise of ‘quick farming’ in peri-urban Uganda. J Biosoc Sci 2023; 55: 995–1014. 10.1017/S0021932023000019 36762463

[51] Schneider S, Salm F, Vincze S et al Perceptions and attitudes regarding antibiotic resistance in Germany: a cross-sectoral survey amongst physicians, veterinarians, farmers and the general public. J Antimicrob Chemother 2018; 73: 1984–8. 10.1093/jac/dky100 29590400

[52] Brown N, Buse C, Lewis A et al Pathways, practices and architectures: containing antimicrobial resistance in the cystic fibrosis clinic. Health 2021; 25: 196–213. 10.1177/1363459319866894 31387378

[53] Whanpuch P, Perris A, Poompruek P et al Microbes and marginalisation: ‘facing’ antimicrobial resistance in bedridden patients in a peri-urban area of Thailand. SSM Qual Res Health 2024; 6: 100489. 10.1016/j.ssmqr.2024.100489

[54] Sringernyuang L, Poompruek P, Whanpuch P. Antimicrobial Resistance, Urban Life in Thailand and Anthropological Research. Mahidol University, Thailand, London School of Hygiene & Tropical Medicine. 2021. 10.17037/PUBS.04661424

[55] Broom A, Kenny K, Prainsack B et al Antimicrobial resistance as a problem of values? Views from three continents. Crit Public Health 2021; 31: 451–63. 10.1080/09581596.2020.1725444

[56] Craig J, Sriram A, Sadoff R et al Behavior-change interventions to improve antimicrobial stewardship in human health, animal health, and livestock agriculture: a systematic review. PLoS Glob Public Health 2023; 3: e0001526. 10.1371/journal.pgph.0001526 37155592 PMC10166487

[57] Denyer Willis L, Kayendeke M, Chandler CI. The politics of irrationality. Med Anthropol Q 2023; 37: 382–95. 10.1111/maq.12809 37703403 PMC10947286

[58] Pinto Jimenez CE, Keestra SM, Tandon P et al One health WASH: an AMR-smart integrative approach to preventing and controlling infection in farming communities. BMJ Glob Health 2023; 8: e011263. 10.1136/bmjgh-2022-011263 PMC10008318 36882219

[59] Dixon J, Manyau S, Kandiye F et al Antibiotics, rational drug use and the architecture of global health in Zimbabwe. Soc Sci Med 2021; 272: 113594. 10.1016/j.socscimed.2020.113594 33529937

[60] da Silva-Brandao RR, de Oliveira SM, Correa JS et al Coping with in-locus factors and systemic contradictions affecting antibiotic prescription and dispensing practices in primary care–a qualitative One Health study in Brazil. PLoS ONE 2023; 18: e0280575. 10.1371/journal.pone.0280575 36662722 PMC9857971

[61] Albernaz-Gonçalves R, Olmos G, Hötzel MJ. Exploring farmers’ reasons for antibiotic use and misuse in pig farms in Brazil. Antibiotics 2021; 10: 331. 10.3390/antibiotics10030331 33809885 PMC8004152

[62] Wafula FN, Miriti EM, Goodman CA. Examining characteristics, knowledge and regulatory practices of specialized drug shops in Sub-Saharan Africa: a systematic review of the literature. BMC Health Serv Res 2012; 12: 223. 10.1186/1472-6963-12-223 22838649 PMC3520114

[63] Olaru ID, Chingono RMS, Bottomley C et al The effect of a comprehensive typhoid conjugate vaccine campaign on antimicrobial prescribing in children in Harare, Zimbabwe: a mixed methods study. Lancet Glob Health 2023; 11: e1422–31. 10.1016/S2214-109X(23)00319-4 37591588 PMC7616073

[64] Yadav H, Shah D, Sayed S et al Availability of essential diagnostics in ten low-income and middle-income countries: results from national health facility surveys. Lancet Glob Health 2021; 9: e1553–60. 10.1016/S2214-109X(21)00442-3 34626546 PMC8526361

[65] Wasan H, Reeta KH, Gupta YK. Strategies to improve antibiotic access and a way forward for lower middle-income countries. J Antimicrob Chemother 2024; 79: 1–10. 10.1093/jac/dkad291 38008421

[66] Årdal C, Balasegaram M, Laxminarayan R et al Antibiotic development—economic, regulatory and societal challenges. Nat Rev Microbiol 2020; 18: 267–74. 10.1038/s41579-019-0293-3 31745330

[67] Nayiga S, Denyer Willis L, Staedke SG et al Taking opportunities, taking medicines: antibiotic use in rural eastern Uganda. Med Anthropol 2022; 41: 418–30. 10.1080/01459740.2022.2047676 35324360 PMC10040720

[68] Nayiga S, Denyer Willis L, Staedke SG et al Reconciling imperatives: clinical guidelines, antibiotic prescribing and the enactment of good care in lower-level health facilities in Tororo, Uganda. Glob Public Health 2022; 17: 3322–33. 10.1080/17441692.2022.2045619 35220900 PMC10083044

[69] Nayiga S, Kayendeke M, Nabirye C et al Use of antibiotics to treat humans and animals in Uganda: a cross-sectional survey of households and farmers in rural, urban and peri-urban settings. JAC Antimicrob Resist 2020; 2: dlaa082. 10.1093/jacamr/dlaa082 34223037 PMC8210029

[70] Nayiga S, MacPherson EE, Mankhomwa J et al ‘Arming half-baked people with weapons!’ Information enclaving among professionals and the need for a care-centred model for antibiotic use information in Uganda, Tanzania and Malawi. Glob Health Action 2024; 17: 2322839. 10.1080/16549716.2024.2322839 38441912 PMC10916894

[71] Chandler CIR . Current accounts of antimicrobial resistance: stabilisation, individualisation and antibiotics as infrastructure. Palgrave Commun 2019; 5: 53. 10.1057/s41599-019-0263-4 31157116 PMC6542671

[72] Mackenbach JP . The contribution of medical care to mortality decline: McKeown revisited. J Clin Epidemiol 1996; 49: 1207–13. 10.1016/S0895-4356(96)00200-4 8892485

[73] Vallin J, Meslé F. Convergences and divergences in mortality. Demogr Res 2004; 2: 11–44. 10.4054/DemRes.2004.S2.2

[74] Permanyer I, Bramajo O. The race between mortality and morbidity: implications for the global distribution of health. Popul Dev Rev 2023; 49: 909–37. 10.1111/padr.12582

[75] Kirchhelle C . Pharming animals: a global history of antibiotics in food production (1935–2017). Palgrave Commun 2018; 4: 96. 10.1057/s41599-018-0152-2

[76] Van Boeckel TP, Pires J, Silvester R et al Global trends in antimicrobial resistance in animals in low- and middle-income countries. Science 2019; 365: eaaw1944. 10.1126/science.aaw1944 31604207

[77] Tang KL, Caffrey NP, Nóbrega DB et al Restricting the use of antibiotics in food-producing animals and its associations with antibiotic resistance in food-producing animals and human beings: a systematic review and meta-analysis. Lancet Planet Health 2017; 1: e316–27. 10.1016/S2542-5196(17)30141-9 29387833 PMC5785333

[78] Just Transitions for AMR Working Group . A just transition for antimicrobial resistance: planning for an equitable and sustainable future with antimicrobial resistance. Lancet 2024; 403: 2766–7. 10.1016/S0140-6736(23)01687-2 37696277

[79] Pokharel S, Adhikari B, Johnson T et al Interventions to address antimicrobial resistance: an ethical analysis of key tensions and how they apply in low- income and middle-income countries. BMJ Glob Health 2024; 9: e012874. 10.1136/bmjgh-2023-012874 PMC11002359 38569658

[80] Bud R . Penicillin: Triumph and Tragedy. Oxford University Press, 2007.

[81] Nerlich B, James R. ‘The post-antibiotic apocalypse’ and the ‘war on superbugs’: catastrophe discourse in microbiology, its rhetorical form and political function. Public Underst Sci 2009; 18: 574–90. 10.1177/0963662507087974 20027773

[82] Servitje L . Medicine is War: The Martial Metaphor in Victorian Literature and Culture. State University of New York Press, 2021.

[83] Hutchison C . Wars and sweets: microbes, medicines and other moderns in and beyond the(ir) antibiotic era. Med Humanit 2022; 48: 359–70. 10.1136/medhum-2021-012366 35948395 PMC9411908

[84] Denyer Willis L, Chandler C. Quick fix for care, productivity, hygiene and inequality: reframing the entrenched problem of antibiotic overuse. BMJ Glob Health 2019; 4: e001590. 10.1136/bmjgh-2019-001590 PMC6703303 31497315

[85] Rabello RF, Bonelli RR, Penna BA et al Antimicrobial resistance in farm animals in Brazil: an update overview. Animals (Basel) 2020; 10: 552. 10.3390/ani10040552 32224900 PMC7222418

[86] Corrêa JS, Zago LF, Da Silva-Brandão RR et al The governance of antimicrobial resistance in Brazil: challenges for developing and implementing a One Health agenda. Glob Public Health 2023; 18: 2190381. 10.1080/17441692.2023.2190381 36934430

[87] Green DL, Keenan K, Fredricks KJ et al The role of multidimensional poverty in antibiotic misuse: a mixed-methods study of self-medication and non-adherence in Kenya, Tanzania, and Uganda. Lancet Glob Health 2023; 11: e59–68. 10.1016/S2214-109X(22)00423-5 36521953

[88] Adams V . Metrics: What Counts in Global Health. Duke University Press, 2016.

[89] Davis MD, Lohm D, Flowers P et al Antibiotic assemblages and their implications for the prevention of antimicrobial resistance. Soc Sci Med 2022; 315: 115550. 10.1016/j.socscimed.2022.115550 36410136

